# Identification of molecular determinants of cell culture growth characteristics of Enterovirus 71

**DOI:** 10.1186/s12985-016-0645-9

**Published:** 2016-11-28

**Authors:** Pinn Tsin Isabel Yee, Kuan Onn Tan, Iekhsan Othman, Chit Laa Poh

**Affiliations:** 1 Research Centre for Biomedical Sciences, Sunway University, Bandar Sunway, Kuala Lumpur, Selangor 47500 Malaysia; 2 Jeffrey Cheah School of Medicine and Health Sciences, Monash University, Bandar Sunway, Malaysia

**Keywords:** Enterovirus 71, Hand foot mouth disease, Pathogenicity, Vaccines, Site directed mutagenesis

## Abstract

**Background:**

Hand, foot and mouth disease is caused by Enterovirus 71 (EV-A71) and Coxsackieviruses. EV-A71 infection is associated with high fever, rashes and ulcers but more severe symptoms such as cardiopulmonary failure and death have been reported. The lack of vaccines highlighted the urgency of developing preventive agents against EV-A71. The molecular determinants of virulent phenotypes of EV-A71 is unclear. It remains to be investigated if specific molecular determinants would affect the cell culture growth characteristics of the EV-A71 fatal strain in Rhabdomyosarcoma (RD) cells.

**Results:**

In this study, several genetically modified sub-genotype B4 EV-A71 mutants were constructed by site-directed mutations at positions 158, 475, 486, 487 and 5262 or through partial deletion of the 5′-NTR region (∆ 11 bp from nt 475 to 486) to generate a deletion mutant (PD). EV-A71 mutants 475 and PD caused minimal cytopathic effects, produced lowest viral RNA copy number, viral particles as well as minimal amount of viral protein (VP1) in RD cells when compared to mutants 158, 486, 487 and 5262.

**Conclusions:**

The molecular determinants of virulent phenotypes of EV-A71 sub-genotype B4 strain 41 (5865/Sin/000009) were found to differ from the C158 molecular determinant reported for the fatal EV-A71 sub-genotype B1 strain (clinical isolate 237). The site-directed mutations (SDM) introduced at various sites of the cDNA affected growth of the various mutants when compared to the wild type. Lowest viral RNA copy number, minimal number of plaques formed, higher infectious doses required for 50% lethality of RD cells and much reduced VP1 of the EV-A71 sub-genotype B4 strain 41 genome was attained in mutants carrying SDM at position 475 and through partial deletion of 11 bp at the 5′-NTR region.

## Background

The World Health Organization (WHO) and the scientific community have been addressing challenges unprecedented in public health posed by Enteroviruses in the post-poliovirus era. Enteroviruses such as Enterovirus 71 (EV-A71), Coxsackie type A16 (CV-A16) and other enteroviruses causing hand, foot and mouth disease (HFMD) have led to over 7 million infections, including 2457 fatalities in China from 2008 to 2012 [1]. However, due to increasing travels and rapid globalization, outbreaks in other parts of the world have also appeared in cyclical epidemics [2, 3].

Within the family *Picornaviridae*, the genus *Enterovirus* comprises 12 species. The species Enterovirus A consists of 25 serotypes and includes the enteroviruses that can cause HFMD such as EV-A71, CV-A16, CV-A5, CV-A6, CV-A8 and CV-A10 [4]. Five years of virological surveillance in China (2008–2014) showed that 43.73, 22.04, and 34.22% of HFMD cases were due to EV-A71, CV-A16 and other enteroviruses, respectively [5]. However, Coxsackie viruses which are common etiological agents of HMFD do not generally cause neurological or cardiopulmonary disease but EV-A71 is the main causative agent of fatal HFMD infections [6]. Mild symptoms of EV-A71 infection in children range from fever (≥39 °C), sore throat, loss of appetite and rash with vesicles on the hands and feet. EV-A71 constituted 80–85% of the pathogens isolated from HFMD-related deaths based on clinical etiological data [7]. The lack of vaccines and antiviral drugs against EV-A71 highlights the urgency and significance of developing preventive and treatment agents against EV-A71.

The EV-A71 virus is classified as Human enterovirus A (HEV-A) species, belonging to the genus *Enterovirus* in the family *Picornaviridae* [4, 8]. Phylogenetic analysis suggests that EV-A71 and CV-A16 were genetically highly related and the common ancestor of EV-A71 is likely to have emerged around 1941 [9]. The EV-A71 virus is a non-enveloped icosahedral viral particle that contains a single-stranded, positive sense, poly-adenylated viral Ribonucleic Acid (RNA) of approximately 7.4 kb. The capsid is made up of 60 protomers, each consisting of 4 polypeptides that comprise the structural proteins, VP1, VP2, VP3 and VP4. Of all the polypeptides, VP4 is located on the internal side of the capsid while VP1, VP2 and VP3 are located on the external surface of the EV-A71 virus [10].

The EV-A71 genome comprises a 5′ non-translated region (5′-NTR), a long open reading frame (ORF) and a short 3′-NTR followed by a poly-adenylated (poly A) tail. The 5′-NTR contains an internal ribosome entry site (IRES) which allows viral protein translation in a cap-independent manner [11]. The ORF is translated into a single large polyprotein of approximately 2100 amino acids (aa) which is divided into three regions (P1-P3). The polyprotein undergoes a series of processing events, culminating in the maturation cleavage of the polyprotein which generates structural and non-structural viral proteins [12]. The four structural proteins, VP1, VP2, VP3 and VP4, are encoded by the P1 region which constitutes the virus capsid. Proteins derived from the non-structural P2 (2A^pro^, 2B, 2BC, 2C^ATPase^) and P3 (3A, 3AB, 3B, 3C^pro^, 3CD^pro^, 3D^pol^) regions are mostly directly involved in viral replication and structural and biochemical changes which are observed within the infected cell [13]. Non-structural proteins (2A and 3C proteinases) are responsible for apoptosis of infected cells in vitro [14, 15].

Identification of molecular determinants in EV-A71 by comparing genome differences have been reported. Li et al. [16] evaluated the sequences of virulent and non-virulent strains and concluded that four amino acids in the capsid protein VP1 (Gly^P710^/Gln ^P710^/Arg ^P710^/Glu ^P729^), Lys ^P930^ in protease 2A and four nucleotides in the 5′-NTR region (G^P272^, U^P488^ and A^P700^/U^P700^) are likely to contribute to the EV-A71 virulent phenotype. The variation of amino acids/nts. at these positions may influence the EV-A71 viral RNA translation efficiency or the enzymatic activity of proteases. Arita et al. [17] isolated an EV-A71 (S1-3′) mutant derived from the EV-A71 genotype A BrCr strain by introducing mutations in the 5′-NTR region (A485G), 3D polymerase (Tyr^P73^, Cys^P363^) and in the 3′-NTR (A7409G) of the EV-A71 genome. However, the virus still caused tremors in 3 cynomolgus monkeys and was isolated only from the spinal cord, indicating that a cooperative effect of the mutations introduced had reduced neuro-virulence of EV-A71 [17, 18].

Yeh et al. [19] constructed a series of infectious clones containing chimeric 5′-NTR regions and mapped a genetic determinant of EV-A71 virulence to nt. 158 within a fragment comprising 12 nucleotides in the stem loop (SL) II structure of EV-A71 clinical isolate 237. A single nucleotide change from cytosine to uridine at position 158 (USLIIM-C158U) caused an alteration in the RNA secondary structure of SL II which led to reduced viral translation and virulence in mice. Mice infected with the USLIIM-C158U mutant was observed to have prolonged average survival time and survival rate as compared with infection with the wild-type USLIIM (40% vs. 0%, *P* < 0.005) [19]. In this study, we are investigating if the EV-A71 sub-genotype B4, strain 41 (5865/Sin/000009) has the same or a different virulence determinant from sub-genotype B1 (clinical isolate 237) and the BrCr prototype strain [17]. The EV-A71 sub-genotype B4 virus (accession number: AF316321) was modified to carry a single mutation at nucleotide position 158 by site-directed mutagenesis to assess if the nucleotide is a common virulence determinant between EV-A71 sub-genotypes B4 and B1.

**Fig. 1:**
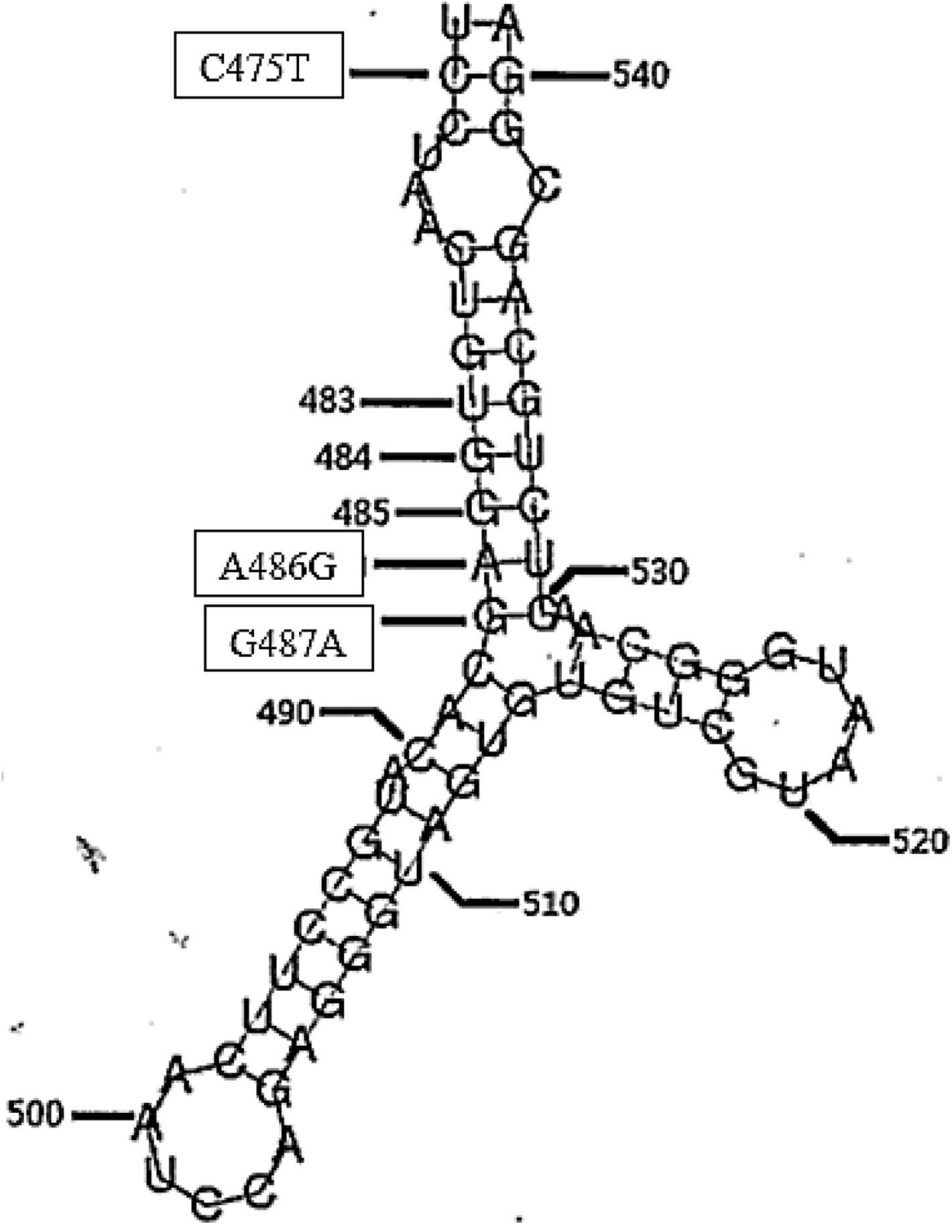
Predicted RNA secondary structure of EV-A71 strain 41 from nt. 474 to 541. Due to slight differences in the length of the 5′-NTR, nt. 480 in Sabin 1 is at the equivalent position 486 in this figure. Nt. 481 in Sabin 2 is at the equivalent position 487, and nt. 472 in Sabin 3 is at the equivalent position 475 of the EV-A71 genome

In addition, we genetically modified the EV-A71 sub-genotype B4 virus by substituting a single nucleotide at either position 475, 486, or 487 based on the fact that the corresponding nucleotides are responsible for the neuro-virulence of Poliovirus Sabin strains 1, 2 and 3, respectively as well as the EV-A71 BrCr prototype strain. Figure 1 illustrates the predicted RNA secondary structure of EV-A71 strain 41 from nt. 474 to 541. There is only a single nucleotide difference between the fatal (GenBank: AF316321) and the non-fatal strain (GenBank: AF352027) isolated from the Singapore outbreak in 2000 based on comparative sequence analysis [20]. The single nt difference at position 5262 in the 3A non-structural region could be the molecular determinant of virulence in the EV-A71 genome. The growth characteristics of the various mutated EV-A71 strains were evaluated in Rhabdomyosarcoma (RD) cell culture by plaque forming ability, viral infectivity by tissue culture infectious dose (TCID_50_) determinations, RNA copy number and the quantification of VP1 formed.

## Results

### Cytopathic effects (CPE) caused by mutants

**Fig. 2:**
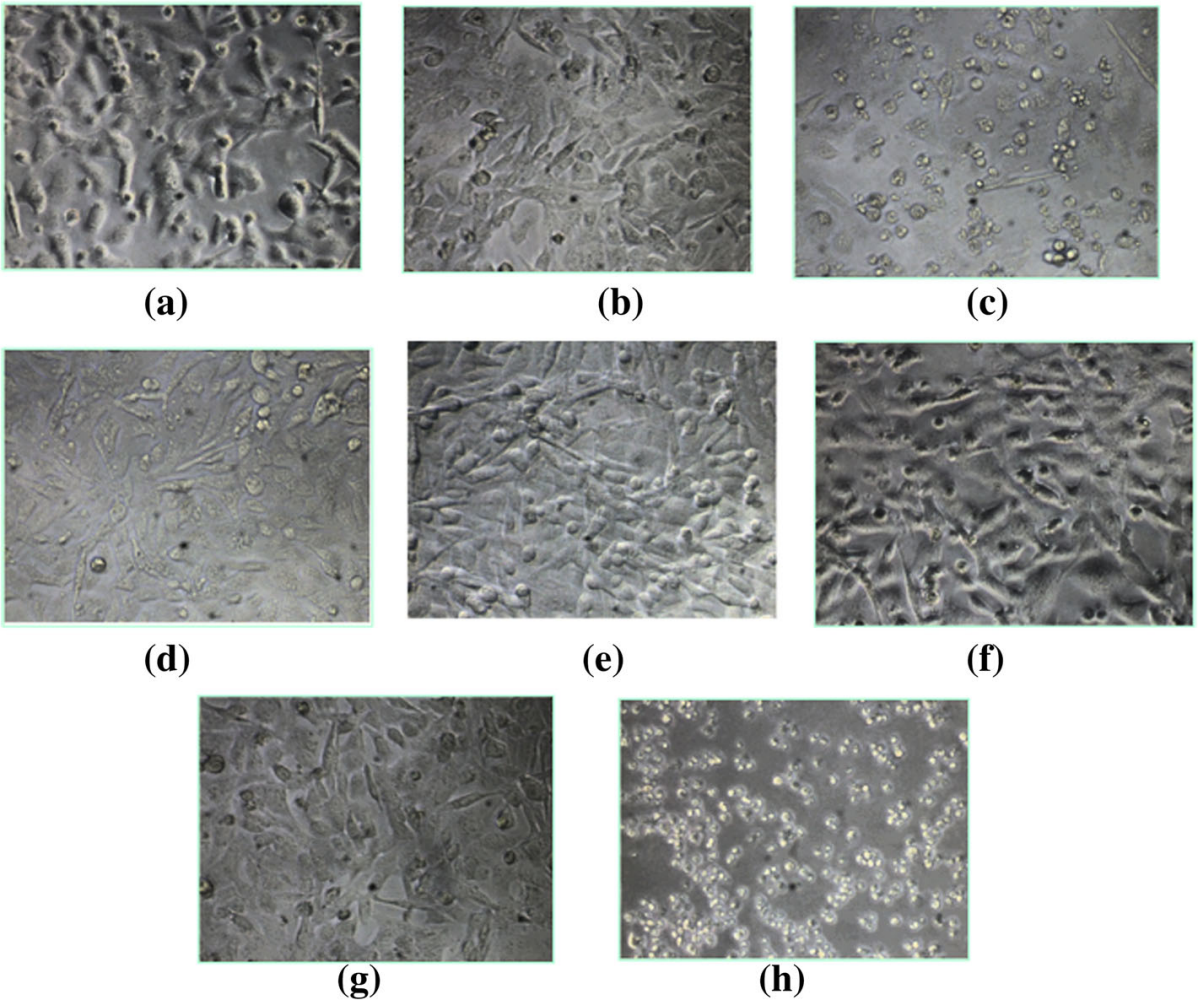
Cytopathic effects (CPE) caused by mutants **a** EV-A71 (A158T) **b** C475T **c** A486G **d** G487A **e** A5262G and **f** PD (Partial Deletant, 5′-NTR) in Rhabdomyosarcoma (RD) cells in comparison with **g** uninfected RD cells (negative control using Opti-MEM) and **h** EV-A71 wild type strain 41 infected RD cells (positive control). Transfection of infectious RNA into RD cells was performed with the use of Lipofectamine 2000 reagent using EV-A71 mutants at a MOI of 0.1. CPE was seen as round and shrunken cells that eventually dislodged from the surface. Magnification used (X 100)

The EV-A71 sub-genotype B4 virus (accession number: AF316321) was genetically modified to carry a single mutation at nucleotide position 158 by site-directed mutagenesis to assess if the nucleotide is a common virulence determinant between EV-A71 sub-genotypes B4 and B1. The effect of this change was evaluated by qualitative growth assays such as cytopathic effects (CPE) in Rhabdomyosarcoma (RD) cells. Mutant EV-A71 158 (A158T) virus showed minimal CPE when transfected into RD cells with a multiplicity of infection (MOI) of 0.1 (Fig. 2a) as compared to the positive control (EV-A71 wild type strain 41) (Fig. 2h). CPE was seen as round and shrunken cells which floated on the surface and there was extensive CPE seen with the EV-A71 wild type strain 41. All experiments were repeated at least three times on separate days.

The virulence determinants of wild type Poliovirus and EV-A71 genotype A BrCr prototype strain reported in previous studies served as references in this study to produce EV-A71 mutants that have reduced virulence in cell cultures. The EV-A71 strain 41 virus was also genetically modified by substituting nucleotides at position 475, 486, or 487 as the corresponding nucleotides were reported to be responsible for neuro-virulence of Poliovirus serotypes 1, 2 and 3, respectively. The mutant EV-A71 475 (C475T) caused the lowest CPE in RD cells (Fig. 2b) when compared to mutants 486 (A486G) (Fig. 2c) and 487 (G487A) (Fig. 2d). Out of the three mutants, A486G caused the highest amount of CPE (Fig. 2c) in RD cells as evident from the rounding and shrunken nature of numerous cells. EV-A71 mutant 487 (G487A) displayed intermediate CPE when transfected into RD cells with a MOI of 0.1 (Fig. 2d).

Analysis of the genomic sequences of two EV-A71 strains, one isolated from a non-fatal case and another isolated from a fatal case shows that a single nucleotide (nt) difference between the fatal strain (EV-A71 strain 5865/Sin/000009, designated as strain 41), and the non-fatal strain (EV-A71 strain 5666/Sin/002209, designated as strain 10) might be responsible for virulence in the fatal strain. This is based on a previous observation by Singh et al. [20] where they indicated the difference between the fatal and non-fatal strain is at nucleotide (nt) position 5262. In the current investigation, the Adenine residue at position 5262 present in the fatal strain (strain 41) was changed to the Guanine residue. Based on the morphology shown in Fig. 2e, infection of RD cells by mutant A5262G showed no CPE as cells were relatively healthy when compared to the extensive lysis observed with the positive wild type control (Fig. 2h). There was hardly any floating cells observed and this is similar to no CPE being observed with the negative control (without virus) (Fig. 2g).

An EV-A71 mutant that carried a partial deletion in the 5′-NTR region in the viral genome was also constructed. This was carried out to reduce the efficiency of viral replication as a deletion in this 5′-NTR region was found to generate genetically stable Poliovirus [21]. In this study, deletion was created from nt. positions 475 to 485 (∆11). The mutant EV-A71 PD (Partial Deletant 5′-NTR) did not show any CPE (Fig. 2f) and the microscopic image showed similarity with the image taken from the uninfected RD cells that acted as a negative control (Fig. 2g). These microscopic images are in direct contrast to the extensive CPE observed for the EV-A71 wild type strain 41 (Fig. 2h).

### Quantification of viral RNA copy number

Observation of CPE in Rhabdomyosarcoma (RD) cells is a qualitative indication of the extent of infectivity of the various mutants in comparison with the EV-A71 wild type strain 41. Approaches such as determination of the viral RNA copy number, plaque forming units (PFU), 50% tissue culture infectious doses (TCID_50_) and amount of viral capsid protein 1 (VP1) present in each strain will enable a better quantitative comparison of the growth characteristics of the various mutant strains. The viral RNA copy number of the various EV-A71 mutant strains was evaluated after growing in Rhabdomyosarcoma (RD) cell culture.

**Fig. 3:**
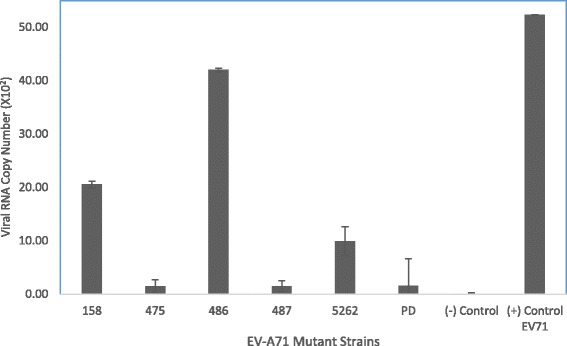
Quantification of Viral RNA Copy Number. Transfection of infectious RNA into RD cells was performed with the use of Lipofectamine 2000 reagent and EV-A71 mutants at a MOI of 0.1. The viral RNA copy number was determined at 24 h post-infection by TaqMan Real-Time PCR. Viral RNA copy numbers are the average of three biological replicates. Error bars represent the standard deviation of the mean

The wild type EV-A71 strain 41 gave the highest yield of viral RNA copy number (5.2 × 10^3^) in RD cells. As for EV-A71 mutants 475 and PD, very low RNA copy numbers were detected for both mutants at 1.02 × 10^2^ and 1.05 × 10^2^, respectively (Fig. 3). There are a few positions that appear to reduce the replication of the virus when the mutated strains were evaluated in vitro (Fig. 3). For example, EV-A71 mutant 158 (A158T) mutated at position 158 gave a yield of 2.0 × 10^3^ RNA copy number which is less than half that of the wild type RNA copy number (5.2 × 10^3^). Amongst all the mutants being evaluated, mutant 486 (A486G) carrying SDM at position 486 expressed the highest viral RNA copy number of 4.2 × 10^3^ when compared to the wild type strain 41. The mutant 487 (G487A) carrying SDM at position 487 produced a viral RNA copy number of 2.0 × 10^2^. The mutant 5262 (A5262G) with a SDM at position 5262 also showed much reduced viral RNA copy number of 7.0 × 10^2^ (Fig. 3).

The mutation introduced at position 5262 did considerably reduce the viral RNA copy number to 10-fold less when compared to the wild type copy number. Therefore, mutant 5262 will need to be further evaluated by plaque forming ability, TCID_50_ and the amount of VP1 formed.

### Infectivity of mutant viruses quantified by tissue culture infectious dose (TCID_50_)

The tissue culture infectious dose (TCID_50_) for the EV-A71 wild type strain 41 and mutant viruses were quantitatively determined. TCID_50_ refers to the quantity of virus that produced a cytopathic effect in 50% of the cultures inoculated. The EV-A71 mutant 158 (A158T) at position 158 demonstrated a higher TCID_50_ of 1.00 × 10^4^ when compared with the positive control EV-A71 wild type strain 41 (TCID_50_ of 1.00 × 10^3^) (Table 1). This indicates that EV-A71 mutant 158 would require a higher quantity of virus (approximately 10 times more) to produce a cytopathic effect in 50% of the cultures inoculated.

**Table 1:** Tissue Culture Infectious Dose_50_ (TCID_50_) of EV-A71 mutants in comparison with the wild type EV-A71 strain 41

EV-A71 Mutants	TCID_50_
A158T	1.00 × 10^4^
C475T	1.00 × 10^6^
A486G	1.00 × 10^4^
G487A	7.50 × 10^5^
A5262G	3.41 × 10^5^
Partial Deletant (PD) 5′-NTR	1.00 × 10^6^
Positive Control (EV-A71 strain 41)	1.00 × 10^3^

RD cells were infected with the EV-A71 wild type and mutant viruses at a MOI of 0.1. The TCID_50_/ml values are calculated using the Reed and Muench formula determined from two independent experiments [26]

In addition, the mutant carrying SDM at position 486 (A486G) also had 10 times increased TCID_50_ value (1.00 × 10^4^) when compared with the positive control EV-A71wild type strain 41, suggesting that the mutant had decreased infectivity. Both mutants 5262 and mutant 487 showed more than 100-fold increase in TCID_50_ value when compared against the EV-A71 wild type strain 41. Mutant 475 with SDM at position 475 and mutant PD both showed a significant 1000 fold increase in TCID_50_ to 1.00 × 10^6^ (Table 1). Analysis of the TCID_50_ values indicates that mutant 475 and the partial deletant (PD) required much higher doses of viruses to cause cytopathic effects in 50% of the tissue cultures.

### Quantification of virus by the plaque assay

**Fig. 4:**
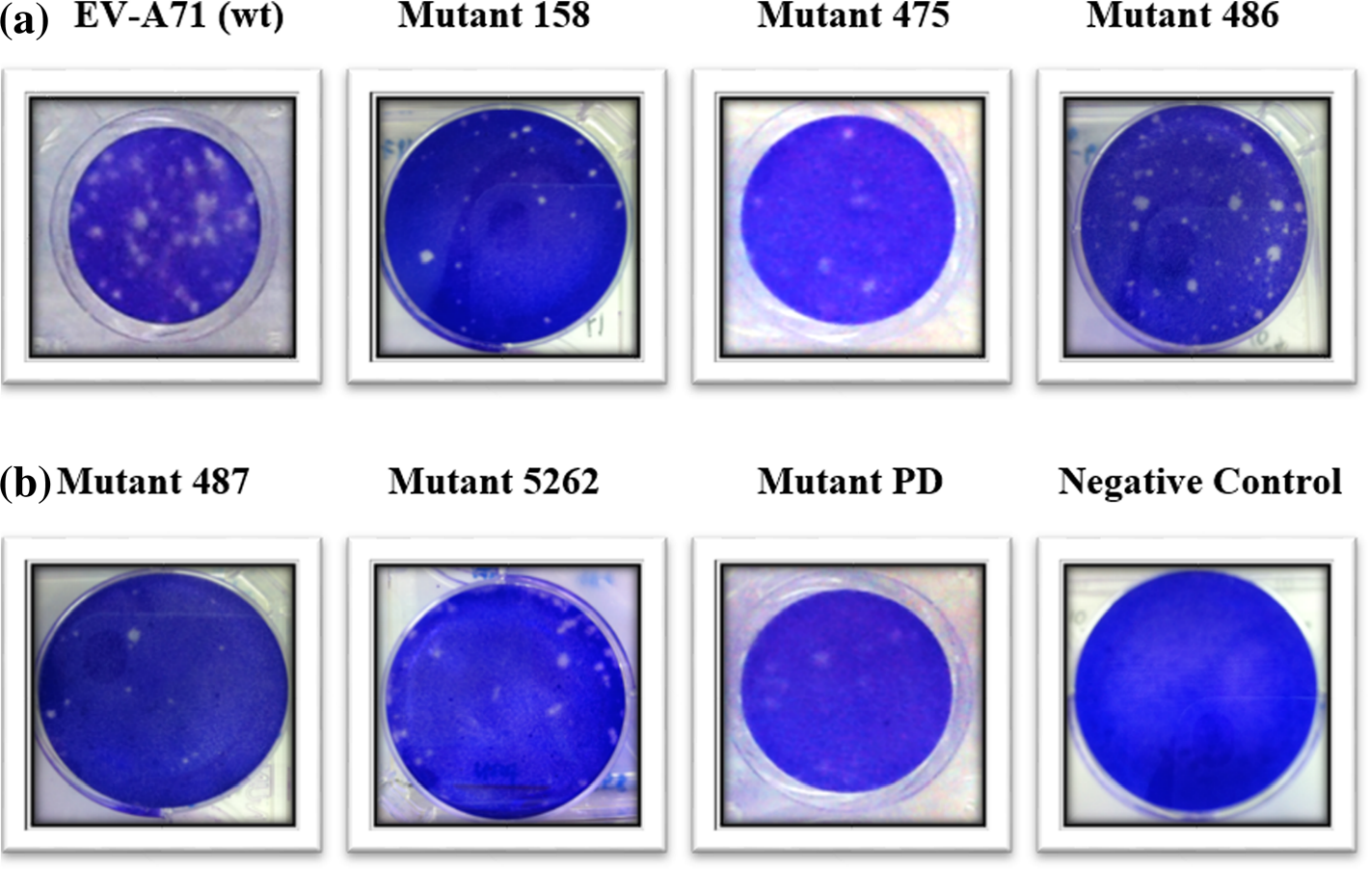
Quantification of plaque forming units by EV-A71 mutants 158, 475, 486, 487, 5262, partial deletant (PD) and the wild type EV-A71 strain 41. The negative control represents uninfected RD cells. EV-A71 mutants were transfected at a MOI of 0.1. The plaque assays were performed on monolayer RD cells incubated at 37 °C and were repeated at least two separate times

**Fig. 5:**
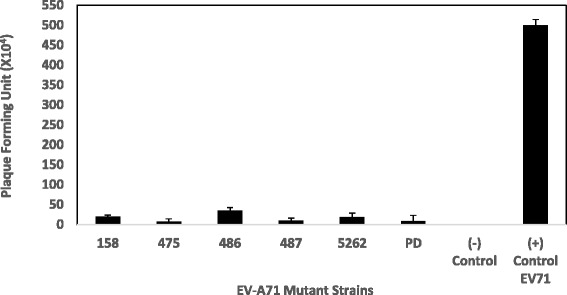
Plaque Forming Units by EV-A71 mutants and the wild type EV-A71 strain 41. RD cells were transfected with EV-A71 mutants and the wild type EV-A71 strain 41 at a MOI of 0.1. Plaque formation was observed 72 h post-infection. PFU numbers are the average of two biological replicates; Error bars represent the standard deviation of the mean

The ability of various EV-A71 mutant strains to form plaques was evaluated in Rhabdomyosarcoma (RD) cell culture (Fig. 4). The wild type EV-A71 strain 41 produced the highest number of plaques (5.0 × 10^7^ PFU/mL) in RD cells. The least number of viral plaques were formed by mutant 475 carrying a site directed mutation (SDM) at position 475 (7.0 × 10^4^ PFU/mL) and the partial deletant (PD) carrying a 11 bp deletion in the 5′-NTR (9.0 × 10^4^ PFU/mL) (Fig. 5). From analysis of the data presented in Fig. 5, there are a few positions in the viral genome that are likely to reduce the ability of the virus to replicate and form plaques. The EV-A71 mutant 158 (SDM at position 158) gave a yield of 2.0 × 10^5^ PFU/ml and the mutant 486 carrying SDM at position 486 also had reduced plaque number (3.5 × 10^5^ PFU/ml) when compared to the positive control (EV-A71 wild type strain 41), albeit at a much higher copy number than the mutant 487 carrying SDM at position 487 (1.0 × 10^5^ PFU/mL).

The mutant 5262 with a SDM at position 5262 also showed much reduced plaque number (1.8 × 10^5^ PFU/mL). As the ability to form plaques is reduced for certain mutant strains, viral growth is expected to be lower. This would mean that viraemia caused by a lower number of viruses will be milder as the viral load would affect virulence of the virus.

### Production of VP1 by mutant strains

**Fig. 6:**
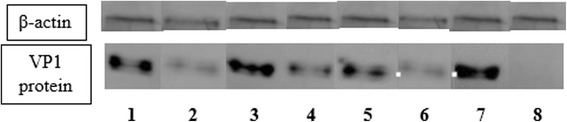
Western blot analysis of VP1 formed by the various EV-A71 mutants. The amount of EV-A71 VP1 protein and β-actin loaded in each lane was 5 μg. The lanes are labelled as follows: lane 1, EV-A71 Mutant 158; lane 2, EV-A71 Mutant 475; lane 3, EV-A71 Mutant 486; lane 4, EV-A71 Mutant 487; lane 5, EV-A71 Mutant 5262; lane 6, EV-A71 Mutant Partial Deletant (PD) 5′-NTR; lane 7, Positive control (EV-A71 Virus); lane 8, Negative control (uninfected RD cells). The molecular weight of the EV-A71 VP1 protein is estimated to be 32.7 kDa with reference to molecular weight markers (from 10–250 kDa) (data not shown)

The ability of the various mutated EV-A71 strains to produce viral particles could be evaluated by detection of VP1 by immunoblotting with the monoclonal antibody directed at VP1. Supernatant derived from transfected RD cells was processed and subjected to electrophoresis to separate the EV-A71 total proteins. The amount of VP1 present in each mutant was assessed by Western blot analysis. A single band was observed at an approximate molecular weight (MW) of 32 kDa (Fig. 6). This band corresponds to the VP1 monomer with an apparent MW of 32.7 kDa.

RD cells infected with the wild type EV-A71 strain 41 (Lane 7) demonstrated the largest amount of VP1 protein (32.7 kDa) being detected in the Western blot analysis. Minimal amount of VP1 was detected in the mutant strain 475 carrying the site specific mutation at position 475 (Lane 2) and the partial deletant (PD) in the 5′-NTR (Lane 6) (Fig. 6). This was consistent with results from plaque numbers, TCID_50_ infectious dose, and RNA copy numbers. There are a few nucleotide (nt) positions in the viral genome that are likely to reduce the infectivity of the virus in RD cells as we observed the amount of viral capsid VP1 being formed was much lesser in some mutants than the amount of VP1 being formed by the wild type strain 41. For example, EV-A71 mutant 158 at position 158 (Lane 1) and mutant 5262 (Lane 5) showed reduced amounts of viral VP1 protein when compared to the wild type EV-A71 (Lane 7) (Fig. 6).

## Discussion

The molecular basis of virulence in EV-A71 is still uncertain. Arita et al. [17] isolated an EV-A71 mutant (S1-3′) carrying mutations in the 5′-NTR region (A485G), 3D polymerase (Tyr-73, Cys-363) and in the 3′-NTR (A7409G) region in the EV-A71 genotype A genome. Although the virulence of the mutant (S1-3′) was significantly reduced, it was still able to cause tremors in cynomolgus monkeys [17]. In addition, Yeh et al. [19] reported that the virulence determinant in an EV-A71 sub-genotype B1 (clinical isolate 237) was due to the presence of cytosine at position 158. This mutation was observed to decrease the virulence of EV-A71 sub-genotype B1 strain [19]. This study demonstrated that the mutation at 158 did reduce viral infectivity by expressing less than half the viral RNA copy number when compared to the wild type but the reduction of infectivity was significantly lower than that observed in EV-A71 mutants 5262, 487, 475 and the partial deletant, PD. Hence, the results show that EV-A71 sub-genotype B4 has a molecular determinant of cell culture growth phenotype which is different from the molecular determinants present in the sub-genotype B1 strain.

In this study, we genetically modified the EV-A71 sub-genotype B4, strain 41 (5865/Sin/000009) by substituting nucleotides at positions 475, 486, and 487 based on the fact that these nucleotides were responsible for the neurovirulence of poliovirus Sabin strains 1, 2 and 3, respectively. From the analysis of nucleotide (nt) sequences present in the three poliovirus Sabin strains, nt. substitutions which were critical in attenuating mutations in the virulent strains isolated from the cerebrospinal fluid were identified. There were 57 nt. substitutions distinguishing the Sabin 1 strain from its parent wild type strain [22]. Among these nt. substitutions, the A480G in the IRES is the most important determinant of the attenuated phenotype of Sabin 1. Their study strongly suggested that nt. 480 influences the formation of a highly ordered structure in the 5′-NTR that is responsible for neuro-virulence [23]. Four other nt. substitutions contributing to the attenuated phenotype were mapped to the capsid region. In addition, there was also one substitution that contributed to the temperature-sensitive phenotype mapped to the 3D^Pol^ region [24].

However, there were only 2 nt. substitutions found in the Sabin 2 strain that appeared at position 481 within the IRES region and at position 2909 within the VP1. For Sabin 3, a total of 10 nt. substitutions were found to differ from its parent strain, but only 3 substitutions appeared to be the main determinants for the attenuated phenotype (C274U in IRES, C2034U in VP3, and U2493C in VP1) [25]. Sabin 3 strain was also found to be the most genetically unstable of the three Sabin strains. As a result of the analysis of the molecular determinants of cell culture growth, in vitro construction of polioviruses with reduced-virulence could be performed via the introduction of mutations in the 5′-NTR to reduce the efficiency of viral replication. The EV-A71 strains had 46% amino acid identity with the polioviral P1 capsid region and 55% with the entire polyprotein. In addition, EV-A71 and PV share high sequence homology especially in the 5′-NTR region [22]. For example, EV-A71 carried the same C nt. at position 475 which corresponds to position 472 in the PV genome.

Li et al. [16] found that there were nine amino acid substitutions (H22Q, P27S, N31S/D, E98K, E145G/Q, D164E, T240A/S, V249I, A289T) that were detected after aligning the VP1 sequences of fatal and mild EV-A71 strains of sub-genotype C4. As such, these residues may be potential virulence determinants in VP1 and could convert mild EV-A71 strains into fatal strains. The study was consistent with previous investigations by Chang et al. [26] who observed that the E145Q substitution at the 5′-NTR was a common difference in the EV-A71 genome isolated from mild and fatal cases of HFMD. Additionally, they also discovered that strains isolated from patients with fatal outcomes had greater substitutions in the 5′-NTR and the IRES regions [26]. This is to be expected as the 5′-NTR was responsible for cap-independent translation of viral proteins. The position of a certain specific amino acid in the genome may have a profound significance on virulence [27]. For example, it was reported that the presence of Asn at position 165 of VP2 in the Coxsackievirus B3 was responsible for a cardiovirulent phenotype [28] or virulence could be due to the cooperative effect of several critical amino acids.

The virulence determinants of Poliovirus and EV-A71 reported in previous studies served as references in this study to produce EV-A71 viruses that are have reduced virulence in cell cultures. An EV-A71 mutant bearing a long deletion in the 5′-NTR of the genome was constructed. In addition, the single nt. difference between the fatal (strain 41) and non-fatal strain (strain 10) of EV-A71 (sub-genotype B4) was examined for virulence by changing the nt. substitution at position 5262 in the EV-A71 viral genome [20]. The mutant 5262 did not completely eliminate the ability to replicate and form plaques. There was some reduction in viral replication by SDM at this site but the effect was less than the effects brought about by introducing SDM into sites 475, 487 and the partial deletion created in the 5′-NTR region. Analysis of the TCID_50_ values indicated that mutants 475 and the PD strain required higher doses of virus to cause cytopathic effects in 50% of the tissue culture. This was consistent with the fact that both mutants have lower infectivity in RD cells than mutants 158, 486, 487, and 5262.

The ability to form plaques was observed to have been reduced for some of the mutants. The virulence of the mutants could be indicated by plaque forming units/ml (PFU/ml). Consistent with the TCID_50_ values, the least number of viral plaques was formed by the mutant carrying SDM at position 475 (7.0 × 10^4^ PFU/mL) and the partial deletant (PD) (9.0 × 10^4^ PFU/mL). The lower PFU/ml indicates that viral growth was slower for these 2 mutants. In addition, RD cells infected with the wild type EV-A71 strain 41 demonstrated the largest amount of the VP1 protein being produced as evident from a thick, single band with molecular weight (MW) of 32 kDa. This band corresponds to the VP1 monomer with an apparent MW of 32.7 kDa. Small amounts of VP1 were produced by mutants 487 and 5262. Mutants 158 and 486 were found to still produce significant amounts of VP1. Minimal amounts of VP1 were detected in the mutant strain carrying the site specific mutation at position 475 and the partial deletant (PD) in the 5′-NTR. The analysis of VP1 indicates that SDM at position 475 and deletion of 11 bp in the 5′-NTR were responsible for significant reduction of infectivity in these 2 strains, respectively.

RD cells infected with the wild type EV-A71 strain 41 gave the highest yield of viral RNA copy number (4.6 × 10^3^). As for EV-A71 mutants 475 and PD, minimal RNA copy number was detected for both mutants at 1.02 × 10^2^ and 1.05 × 10^2^ viral RNA, respectively. This was consistent with the minimal number of plaques being formed, higher infectious doses required for 50% lethality of RD cells and much reduced VP1 being observed. Amongst all the mutants being evaluated, mutant 486 carrying SDM at position 486 (A486G) expressed the highest viral RNA copy number of 4.2 × 10^3^ when compared to the wild type strain 41.

## Conclusions

Mutant 158 was found *not* to have the most reduction in infectivity of RD cells in vitro as it still produced more RNA, viral particles and VP1 than mutants 5262, 487, 475 and PD, it can be inferred that it is not the single virulence determinant for strain 41 which belongs to the sub-genotype B4. This is in contrast to the findings by Yeh et al. [19] who established that the molecular determinant C158 was responsible for molecular virulence in the sub-genotype B1. The single site mutation created by introducing a nt. change at position 5262 did not completely eliminate the ability to replicate and form plaques in RD cells. There was some reduction of viral growth by SDM at this site but the effect was less than the effects brought about by introducing SDM into either sites 475, 487 or the partial deletion created in the 5′-NTR region.

The data reported in this investigation shows that a partial deletion created in the 5′-NTR and SDM at nt. 475 maybe potential molecular determinants of cell culture growth characteristics of EV-A71 in RD cells. Further studies should be conducted to investigate the stability of each of the mutation that was created and to further explore the role of early (virus attachment) and late events (virus assembly, release) in the pathogenesis and expression of the growth phenotype of EV-A71.

## Methods

### Cell culture

Human Rhabdomyosarcoma cells (RD, ATCC # CCL-136) were cultured in Dulbecco’s modified Eagle’s minimal medium/F-12 (DMEM/F-12, Invitrogen, USA), supplemented with 10% foetal bovine serum (FBS) (Gibco, USA), 1% penicillin/streptomycin, 1% L-glutamine and 1% essential amino acids. The cell line was grown at 37 °C in 5% CO_2_ until 80–90% confluency.

### Viral infection

RD cell monolayers in 75-cm^2^ flasks were inoculated with 100 μL EV-A71 strain 5865/Sin/000009 (GenBank accession number AF316321). Infected cells were incubated with 1 mL DMEM containing antibiotics but no serum at 37 °C until complete cytopathic effect (CPE) was apparent. RD cells were frozen at −80 °C and thawed, three times, and cell debris was removed by centrifugation at 14,000 x g for 10 min at 4 °C. Supernatants were used for harvesting the virus.

### RNA extraction and reverse transcription

Viral RNA was extracted from EV-A71 using the QIAmp® Viral RNA Mini Spin Kit (Qiagen, Calif., USA). The kit was used according to the manufacturer’s instructions. The purified RNA was reverse transcribed into cDNA by using the SuperScript® III First Strand Synthesis SuperMix Kit (Invitrogen, Calif., USA). Each cDNA synthesis mixture contained 10X annealing buffer, 25 mM MgCl_2_, 0.1 M oligo(dT), RNAseOUT/Superscript III RT and viral RNA as template.

### Cloning of EV-A71 cDNA into the pCR-XL-TOPO vector

Full length cDNA of EV-A71 was amplified using polymerase chain reaction (PCR). Each PCR reaction contained 5X Phusion HF buffer, 10 mM dNTP, 0.1 g of each primer, 1 μg template cDNA and Phusion HSII DNA Polymerase. The following cycling conditions were employed for the PCR reactions: 98 °C for 30s, followed by 98 °C for 10s, 64 °C for 30s and 72 °C for 5 min for 30 cycles. The cycles were terminated with a 30s extension at 68 °C. The amplified cDNA was cloned into the pCR®-XL-TOPO® vector (Invitrogen, Calif., USA). Approximately 100 ng of the full length EV-A71 cDNA was ligated to 1 μL pCR®-XL-TOPO® vector according to the manufacturer’s protocol in the TOPO® XL PCR Cloning Kit (Invitrogen, Calif., USA). The ligation mixture was incubated for 5 min at room temperature, then 1 μl of the 6x TOPO® Cloning stop solution was added and the tubes were placed on ice. The recombinant EV-A71 pCR®-XL-TOPO® vector was stored at −20 °C for further downstream processes.

### Site-directed mutagenesis

The EV-A71 full genome in the pCR®-XL-TOPO® plasmid acted as the substrate for single mutations using designed primers and the QuickChange Lightning Site-Directed Mutagenesis Kit (Agilent Technologies, Calif., USA). EV-A71 mutants were constructed by introducing site-directed mutations at nucleotide positions 158, 475, 486, 487, in the 5′-NTR and at position 5262 of the viral genome, respectively. In the 1^st^ stage of the reaction, PCR was carried out. The mixture contained 10x reaction buffer, 100 ng/μl of each primer, 100 ng ds DNA template, 1 μL dNTP mix and 1 μL QuikChange Lightning Enzyme mix. The PCR mixture was added up to a total reaction volume of 50 μl. The PCR conditions were set up at 95 °C for 2min.

In the 2^nd^ stage of mutagenesis, 2 μl of the *Dpn*I restriction enzyme was added to the amplification product to digest the methylated and hemi-methylated parental DNA. The ligation mixture was incubated for 5min at 37 °C. In the 3^rd^ stage, transformation was performed using XL 10-Gold® Ultracompetent cells (Agilent Technologies, Calif., USA). The ligated mixture (2 μl) was added to the competent cells and incubated on ice for 30min. The cells were then subjected to heat shock at 42 °C for 30s, and immediately placed on ice for 2min. An aliquot of the NZY^+^ broth (500 μl) was added to the XL 10-Gold competent cell suspension, followed by shaking of the transformed bacterial cells at 37 °C for 1 h. After the 1 h incubation, tubes were centrifuged at 13,000 × g for 1min. Clear supernatant was discarded and the cell pellet was resuspended in 500 ul of fresh NZY^+^medium and an aliquot of 0.2 ml was plated on LB agar supplemented with 25 μg/ml kanamycin sulphate (Invitrogen, Calif., USA). The *E.coli* cells were incubated overnight for 16 h at 37 °C. Nucleotide substitutions were confirmed by DNA sequence analysis using nucleotide Basic Local Alignment Software (BLAST by NCIB).

### Partial-deletion in the 5′-NTR of EV-A71 genome

EV-A71 with a partial deletion from nucleotide positions 475 to 485 in the 5′-NTR was constructed using two designed primers that were 24 nucleotides in length [namely EV-UTR-F(∆11) and EV-UTR-R(∆11)] and the Q5® Site-Directed Mutagenesis Kit (New England Biolabs, USA) . The EV-A71 deletion mutant was confirmed by DNA sequence analysis by nucleotide Basic Local Alignment Software (BLAST by NCIB). Bacterial colonies that carried the viral genome with the correct mutations were kept in 20% glycerol stock at −80 °C for long term storage.

### Restriction endonuclease digestion of plasmid DNA

Plasmid DNA (1–5 μg) purified from *E.coli* transformants was digested with *Eag*I (New England BioLabs, Massachusetts, USA). The reaction was incubated at 37 °C in a water bath for 2 h. Screening of DNA fragments after digestion of the recombinant pCR-XL-TOPO EV-A71 plasmid was carried out using DNA agarose gel electrophoresis. The agarose gel was pre-stained with GelRed nucleic acid stain (Biotium, USA) prepared in 0.5X TAE buffer. DNA products were mixed with gel loading buffer and loaded into wells in the agarose gel. Electrophoresis was carried out using 80 V and DNA bands were illuminated with UV light. The size of the DNA fragments were compared with a GeneRuler 1 kbp DNA ladder (Invitrogen, USA).

### Phenol-chloroform purification and ethanol precipitation of DNA

The DNA solution was mixed with an equal volume of phenol-chloroform (Invitrogen, Calif., USA) and vortexed for 1min. The mixture was centrifuged at 15,000 × g for 10min to separate the aqueous and solvent phases. The desired aqueous layer was carefully removed and mixed with an equal volume of phenol-chloroform (1:1) (Invitrogen, Calif., USA) and vortexed for 1min. The mixture was again centrifuged at 15,000 × g for 10min. The aqueous layer was removed and 1/10 volume of chilled 3 M sodium acetate (pH 5.2) and 1/10 volume of isopropanol were added to the DNA sample and mixed well by inverting the tube multiple times. Then, the sample was incubated at −20 °C for 2 h to precipitate the DNA. The DNA was pelleted by centrifugation at 15,000 × g for 30min at 4 °C. The supernatant was carefully removed and 1 ml of cold 70% ethanol was added to wash off the remaining salts. The DNA pellet was allowed to air dry for 5min with the lid opened. The desired amount of TE buffer was added into the tube in order to dissolve the DNA

### Production of infectious EV-A71 RNA from cloned cDNA

RNA transcription was carried out using RiboMAX™ Large Scale RNA Production System-SP6 (Promega, Calif., USA). The reaction was set up in 20 μl of reaction volume by following the recommended protocol. The reaction mixture was subjected to incubation at 37 °C for 4 h and followed by DNase treatment for 30min. The DNase (Promega, Calif., USA) was added to the in vitro transcription reaction at a final concentration of 1 U per μg of DNA template. The Linear Control DNA supplied by RiboMAX™ Large Scale RNA Production System-SP6 was used as a template for the production of RNA transcripts of approximately 1.8 kb in length and served as a positive control for the in vitro transcription reaction. The in vitro transcribed EV-A71 RNA was visualized by agarose gel electrophoresis prior to being used in transfection of RD cells [29].

### Transfection of RD cells with in vitro RNA transcripts

RD cells were seeded in a 24-well plate and incubated for 24 h. When the cell confluence reached about 90%, transfection of the RNA transcripts was performed with the use of the Lipofectamine 2000 reagent (Invitrogen, Calif., USA). The transfection reagent was washed away 4 h after transfection and cells were then cultured in fresh medium containing 10% foetal bovine serum. Once cytopathic effects (CPE) were observed (round and shrunken cells), the mutated viruses were harvested.

### Plaque assay

A 6-well plate with 6 × 10^5^ RD cells/well was prepared and incubated overnight at 37 °C in 5% CO_2_. Prior to viral infection, the complete growth medium (DMEM supplemented with 10% FBS) was removed and approximately 1 mL serial 10-fold dilutions of virus inoculum was added to the cells for 1 h at room temperature with gentle shaking to allow for virus attachment. After 1 h incubation, the inoculum was removed and replaced with 2 mL of 1.2% *w/v* carboxylmethylcellulose. After 72 h incubation, the plaque medium was removed and the cells were fixed with 4% formaldehyde and stained with 0.5% crystal violet. The plaques were visible against a white background.

### Tissue Culture Infectious Dose (TCID_50_) assay

The EV-A71 mutant virus titers were quantitatively determined by TCID_50_. TCID_50_ refers to the quantity of a virus that produced a cytopathic effect in 50% of the cultures inoculated. The TCID_50_ assay was carried out in RD cells using the Reed and Muench formula [26]. A monolayer of RD cells from a 75 cm^2^ tissue culture flask was harvested after trypsinization with 1 ml of trypsin-EDTA (Gibco, Calif., USA) and the addition of MEM growth media to obtain a final concentration of 1 × 10^3^ cells /μl. To assay the number of infectious virions in a purified virus stock, serial 10-fold dilutions of the stock virus suspension in quadruplicates, were carried out in a 96-well microtiter plate using MEM growth media as diluent. The negative control wells contained RD cells without any virus. The plate was incubated at 37 °C and observed daily for CPE for up to 48 h.

### Real-time RT-PCR

Total RNA was extracted from various EV-A71 mutant strains grown in RD cells using the RNeasy extraction kit (Qiagen, USA). Quantitative real-time PCR was performed using the Touch^TM^ Real-Time PCR Detection System (Bio-Rad, CFX96), with 4 μl of RNA template, 10 μl of 2x SensiFAST probe No-ROX One Step Mix, 0.8uL Forward and Reverse primer (10uM), 0.2 uL probe (10uM), 0.2 ul reverse transcriptase, and 0.4 uL RiboSafe RNase inhibitor (BioLine, California, USA) in 20 μl of the final reaction mixture. The reaction was performed for one cycle at 48 °C for 10min, 95 °C for 2min, followed by 40 cycles at 95 °C for 5s and 60 °C for 20s in a 96-well plate. Three independent experiments were conducted for each sample. Threshold cycle value (Cq) data was determined using default threshold settings, and the mean Cq was calculated from the duplicate PCRs. A standard graph was plotted based on a series of standard solutions.

### EV-A71 VPl Immunoblot

Total cellular proteins were extracted by incubating wild-type and EV-A71 mutant cells in lysis buffer (20 mM Tris–HCl, pH 7.4, 1% Nonidet P-40, 137 mM NaCl, 50 mM EDTA, protease inhibitor mixture and 1 mM phenylmethylsulfonyl fluoride). After centrifugation at 14,000 × g for 10min at 4 °C, supernatants were transferred and mixed with an equal amount of protein sample buffer (120 mM Tris–HCl, pH 8.0, 20% glycerol, 4% SDS, 2.5% β-mercaptoethanol and 0.05% bromophenol blue). Lysates were separated by SDS-PAGE and transferred to nitrocellulose membrane (Millipore, Calif., USA) that had been blocked with 5% BSA in Tris-Buffered Saline and Tween 20 (TBST) buffer for 1h at room temperature. The membrane was then incubated overnight at 4 °C with anti-enterovirus VP1 monoclonal antibody (LifeSpan BioSciences, Calif., USA) diluted in the blocking buffer. After hybridization with primary antibody, the membrane was washed three times with TBST before hybridizing with anti-mouse HRP secondary antibody (Sigma Aldrich, St Louis, USA) diluted in TBST. The blots were then washed three times with TBST and detected by chemiluminescence using ImageQuant (GE Healthcare Life Sciences, Calif., USA).

## Abbreviations

5′-NTR: 5′- non translated region
CPE: Cytopathic effects
EV-A71: Enterovirus 71
LAV: Live attenuated vaccine
MOI: Multiplicity of infection
nt: Nucleotide
PD: Partial deletant
PFU: Plaque forming units
PV: Poliovirus
RD: Rhabdomyosarcoma
SDM: Site-directed mutations
TCID_50_: 50% tissue culture infectious dose
VP1: Viral protein
